# Growth Hormone Deficiency in an Adolescent With Pseudohypoparathyroidism Type 1B

**DOI:** 10.1210/jcemcr/luae152

**Published:** 2024-08-27

**Authors:** Sabitha Sasidharan Pillai, Monica Reyes, Harald Jüppner, Lisa Swartz Topor

**Affiliations:** Division of Pediatric Endocrinology, Hasbro Children's Hospital, Providence, RI 02903, USA; Department of Pediatrics, The Warren Alpert Medical School of Brown University, Providence, RI 02903, USA; Endocrine Unit and Pediatric Nephrology, Massachusetts General Hospital, Boston, MA 02114, USA; Endocrine Unit and Pediatric Nephrology, Massachusetts General Hospital, Boston, MA 02114, USA; Division of Pediatric Endocrinology, Hasbro Children's Hospital, Providence, RI 02903, USA; Department of Pediatrics, The Warren Alpert Medical School of Brown University, Providence, RI 02903, USA

**Keywords:** pseudohypoparathyroidism type 1B, GHRH resistance, growth hormone deficiency, child

## Abstract

We report growth hormone (GH) deficiency due to presumed GH releasing hormone (GHRH) resistance in an adolescent with pseudohypoparathyroidism type 1B (PHP1B) due to paternal uniparental disomy of chromosome 20 (patUPD20). A male patient aged 11 years 10 months with obesity and mild developmental delay was found to have hypocalcemia, hyperphosphatemia, and an elevated parathyroid hormone level. History included muscle cramps and leg pain with activity. Examination showed round facies, short stature, and obesity. He was in puberty and bone age was advanced by > 2 years. Detailed genetic workup, including nucleotide sequence analysis of *GNAS* exons 1-13 and *STX16*, methylation-sensitive multiplex ligation-dependent probe amplification and analysis of several microsatellite markers for chromosome 20, established the diagnosis of PHP1B due to patUPD20. Muscle cramps and hypocalcemia resolved with calcium carbonate, ergocalciferol, and calcitriol treatment. He was short with linear growth deceleration at around age 13 years. Peak GH concentration was insufficient following stimulation testing. Growth velocity improved with human GH treatment. Although rare, resistance to GHRH can occur in PHP1B and patients with this disorder should be evaluated for GH insufficiency if they present with short stature and reduced growth velocity. Treatment with recombinant human GH may improve growth velocity in such patients.

## Introduction

Albright et al introduced the term pseudohypoparathyroidism (PHP) in 1942 when these investigators described several patients with hypocalcemia and hyperphosphatemia associated with a constellation of several clinical findings, including obesity, short stature, brachydactyly, round face, ectopic ossifications, and neurocognitive impairment. The latter physical findings are referred to as Albright hereditary osteodystrophy (AHO), classically seen in pseudohypoparathyroidism type 1A (PHP1A) (1). In contrast to PHP1A, patients with PHP1B have less obvious or no AHO features; hormone resistance is usually limited to parathyroid hormone (PTH) with some patients showing mild resistance to thyroid-stimulating hormone (TSH) (2). Few PHP1B patients with partial or unquantified growth hormone (GH) deficiency due to presumed GH releasing hormone (GHRH) resistance have been described, including 2 patients with PHP1B due to methylation defects affecting all differentially methylated regions (DMRs) at the *GNAS* locus and monozygotic twins with autosomal dominant PHP1B (AD-PHP1B) due to a *STX16* microdeletion (3-5). Here we report GH deficiency in an adolescent male with PHP1B due to paternal uniparental disomy involving the entire chromosome 20 (patUPD20), a disease cause that has not been previously associated with GH deficiency.

## Case Presentation

A male patient aged 11 years 10 months was referred for incidental detection of hypocalcemia as part of laboratory studies done for evaluation of allergies by his pediatrician. He reported cramps and leg pain with long walks or after trampoline jumping. He preferred to be sedentary. He denied perioral numbness, tingling, seizures, abdominal pain, diarrhea, or vomiting. He ate a varied diet, including meat, and drank up to 1 gallon of cow's milk daily. He did not take medications.

Past medical history included mild deficiency of factor V activity (50% of normal activity) diagnosed at 5 years of age during hospitalization for pneumonia. He had left orchiopexy at 5 years of age. He was born at 38 weeks with a birth weight of 2.86 kg (25%ile-50%ile) and length of 46.5 cm (10%ile-25%ile). His weight was always at the upper end of growth chart with height toward the lower end of normal. Developmental milestones were delayed. He was diagnosed with attention-deficit/hyperactivity disorder (ADHD) and learning impairments, and he receives an individualized education program at school.

The patient is the only child of his parents. There is a history of short stature in multiple family members (mother, 152.4 cm; father, 162.6 cm; maternal grandfather, 152.4 cm; maternal grandmother, 147.3 cm; maternal aunt, 152.4 cm). No family members are known to have calcium disorders, kidney stones, or skeletal abnormalities.

## Diagnostic Assessment

On examination, the patient had short stature and round facies. Ears had simple helices. His weight was 52.5 kg (89%ile, + 1.25 SDS), height 136 cm (4.37%ile, −1.71 SDS) and body mass index 28.37 (98.9%ile, + 2.04 SDS) (5). The upper to lower segment ratio was 0.94. He had Tanner 2 pubic hair and testicular volumes of 15 mL on the right and 10 mL on the left. He did not have obvious skeletal abnormalities, short metacarpals, or ectopic calcifications.

**Figure 1.**
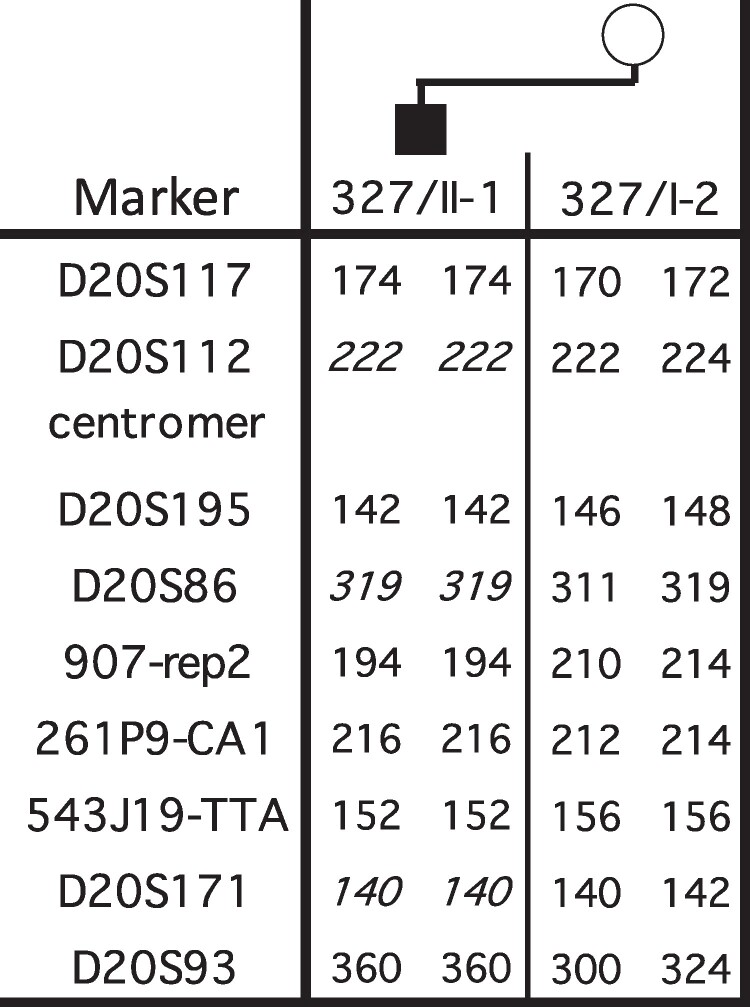
Analysis of different microsatellite markers for chromosome 20q (performed at the DNA Core Facility of the Massachusetts General Hospital, as described by Takatani et al (6)): results are shown for genomic DNA from the patient and his mother; paternal DNA was not available. Bold numbers, fully informative markers; bold italic numbers, partially informative.

Laboratory parameters at presentation are shown in Table 1. His workup suggested a diagnosis of PHP as the unifying explanation for obesity, short stature, round facies, orchiopexy, learning delays, hypocalcemia, and hyperphosphatemia with elevated PTH. Additional studies showed subclinical hypothyroidism with elevated anti-thyroid antibodies. Bone age was read as 14 years, which was advanced by 2 years. He had a normal chromosomal microarray analysis. Nucleotide sequence analysis of *GNAS* and *STX16* revealed no evidence for an abnormal variant. Methylation-sensitive multiplex ligation-dependent probe amplification (MS-MLPA) showed lack of methylation at *GNAS* exons A/B, XL, and AS with increased methylation at exon NESP, while multiplex ligation-dependent probe amplification (MLPA) revealed no evidence of a change in copy number; MLPA and MS-MLPA were performed using the MRC Holland kit ME034, as described (6). These findings were consistent with sporadic PHP1B. Analysis of several microsatellite markers for both arms of chromosome 20 was consistent with patUPD20 (Fig. 1).

**Table 1.** Laboratory parameters of the patient at presentation

Laboratory studies (blood)	At presentation	Reference ranges
Calcium	7.1 mg/dL (1.77 mmol/L)	8.5-10.5 mg/dL (2.12-2.62 mmol/L)
Ionized calcium	3.3 mg/dL (0.82 mmol/L)	4.2-5.2 mg/dL (1.05-1.3 mmol/L)
Phosphorus	7.8 mg/dL (2.52 mmol/L)	3.3-6.2 mg/dL (1.07-2 mmol/L)
Magnesium	1.8 mg/dL (0.74 mmol/L)	1.3-1.9 mg/dL (0.53-0.78 mmol/L)
Alkaline phosphatase	173 IU/L (2.88 μkat/L)	103-373 IU/L (1.72-6.22 μkat/L)
25(OH) vitamin D	11.7 ng/mL (29.2 nmol/L)	30-100 ng/mL (74.88-249.6 nmol/L)
Parathyroid hormone	304 pg/mL (304 ng/L)	18-80 pg/mL (18-80 ng/L)
TSH	8.447 μIU/mL (8.447 mIU/L)	0.35-5.5 μIU/mL (0.35-5.5 mIU/L)
Free T4	0.91 ng/dL (11.71 pmol/L)	0.8-1.8 ng/dL (10.3-23.2 pmol/L)
Anti-thyroid peroxidase autoantibody	238.8 IU/mL (238.8 kIU/L)	1-60 IU/mL (1-60 kIU/L)
Antithyroglobulin autoantibody	94.1 U/mL (94.1 kIU/L)	0-60 U/mL (0-60 kIU/L)
IGF-1	219 ng/mL (28.6 nmol/L)	37-459 ng/mL (4.8-60 nmol/L)
IGFBP-3	5550 ng/mL (5.55 mg/L)	1828-6592 ng/mL (1.82-6.59 mg/L)
Luteinizing hormone	1.1 IU/L (1.1 mIU/mL)	0.06-4.77 IU/L (0.06-4.77 mIU/mL)
Follicle stimulating hormone	2.5 mIU/mL (2.5 IU/L)	0.53-4.92 mIU/mL (0.53-4.92 IU/L)
**Imaging studies**	**Result**	
Bone age x-rays at 11^10^/_12_ y & 12^11^/_12_ y	14 years	—

Abbreviations: IGF-1, Insulin-like growth factor 1; IGFBP-3, Insulin-like growth factor binding protein 3; PTH, parathyroid hormone; T4, thyroxine; TSH, thyrotropin (thyroid-stimulating hormone); y, years.

## Treatment

The patient was treated with elemental calcium, calcitriol, and ergocalciferol. He was also started on levothyroxine after his TSH level rose to 11.6 μIU/mL (reference range, 0.35-5.5 mIU/L). Review of his growth chart showed decreased height percentiles without evidence of a pubertal growth spurt. At 12 years 11 months, his height was 138.9 cm (1.6%ile, −2.15 SDS), growth velocity was 4.1 cm/year, and bone age remained 14 years. GH stimulation using clonidine and arginine showed a peak GH level of 1.5 ng/mL on immunochemiluminometric assay, indicating GH deficiency. Treatment began with nightly injections of recombinant human GH (rhGH) (0.02 mg/kg/day), which was later changed to lonapegsomatropin (0.22 mg/kg/week) due to lack of availability of the daily formulation.

## Outcome and Follow-Up

The patient remained asymptomatic on calcium and vitamin D supplementation. Interval growth velocity after 4 months of rhGH treatment improved to 10.3 cm/year.

## Discussion

The term PHP encompasses disorders characterized by a common defect in agonist-dependent cyclic adenosine monophosphate (cAMP) signaling downstream of different G-protein–coupled receptors, including the PTH/PTH-related peptide receptor (7). Wide variation in presentation and severity can occur among patients with PHP, even with the same genetic mutation (7).

Our patient had AHO features, such as obesity, round facies, and short stature. Although PTH resistance is the most salient feature of PHP1B, AHO features have been reported in rare patients with PHP1B, suggesting clinical overlap among PHP disorders despite different molecular defects (7).

Hormone resistance in PHP1B is usually limited to PTH and TSH, unlike resistance to multiple hormones frequently observed in patients with PHP1A. It is thought that maternal *GNAS* defects that impair the alpha-subunit of stimulatory G protein (Gsα) function are more severe than the epigenetic modifications leading to PHP1B, thus explaining the more modest phenotype in the latter disease (2). Patients with PHP1B often have TSH levels that are at the upper end of normal or slightly elevated, as in our patient (7). He also had detectable anti-thyroid autoantibodies; coexistence of autoimmune thyroiditis and PHP has been reported previously (8).

PHP is characterized by resistance to PTH in proximal tubules of the kidney due to loss or markedly decreased expression of Gsα (6). Gsα is encoded by exons 1 to 13 of *GNAS,* a complex imprinted locus on the long arm of chromosome 20 (20q13.3). Gsα is necessary for mediating the actions of multiple hormones at different G-protein–coupled receptors, including the receptors for PTH, TSH, gonadotropins, and GHRH (6, 9). *GNAS* undergoes parent-specific changes in DNA methylation, limiting expression of several mRNA transcripts derived from this genetic locus to one parental allele (1). Molecular variations at *GNAS* results in a spectrum of phenotypes based on the parental localization of the genetic defect (10). Heterozygous inactivating *GNAS* mutations involving exons 1 to 13 lead to PHP1A when located on the maternal allele and pseudo-PHP or progressive osseous heteroplasia when located on the paternal allele. PHP1B results from imprinting defects at one or several DMRs at the maternal *GNAS* locus (10). PHP1B can be familial or sporadic. Heterozygous maternal microdeletions within *STX16* are the most predominant cause of AD-PHP1B due to loss of methylation restricted to the maternal *GNAS* exon A/B. In contrast, deletions involving the maternal *GNAS* locus cause methylation changes at several DMRs. Patients with sporadic PHP1B frequently have broad epigenetic methylation defects affecting several *GNAS* DMRs, but the underlying genetic defect(s) remains unknown, except for sporadic patUPD20 involving the entire chromosome 20, or portions of the long arm of that chromosome (1).

Our patient was diagnosed with sporadic PHP1B due to patUPD20 based on the analysis of several microsatellite markers located on both arms of chromosome 20 for the patient and his mother. PatUPD20 can be detected in about 10% of cases with sporadic PHP1B, which has significant implications for genetic counseling as the underlying defect will not be passed to the next generation (6).

Unlike in PHP1A, GHRH resistance is rare in patients with PHP1B. Although magnetic resonance brain imaging to assess the pituitary structure was not done in our patient, the most likely reason for his GH deficiency is GHRH resistance. Measurement of serum GHRH level may not have been revealing because of the short half-life of this peptide hormone in the circulation, and unfortunately, synthetic GHRH for diagnostic testing is no longer available. The literature includes descriptions of 4 patients with PHP1B who most likely had GHRH resistance (3-5). Fernández-Rebollo et al reported an adult patient with a height below the 3rd percentile who was diagnosed with PHP1B due to loss of methylation at *GNAS* exons AS, XL, and A/B and gain of methylation at exon NESP, at age 33.2 years. Subsequently, at age 34.2 years he was diagnosed with subclinical hypothyroidism and GH deficiency, although no details regarding the tests leading to the latter diagnosis were provided (3). Mantovani et al reported a 48-year-old male patient with PHP1B due to imprinting defects at the *GNAS* locus (5) with a height of 172 cm (−0.8 SDS), who had a peak GH concentration of 12.3 μg/L (12.3 ng/mL) (reference > 16 μg/L (> 16 ng/mL)) after diagnostic stimulation. However, since GHRH had been combined with arginine for testing, it is uncertain whether his partial GH deficiency was due to resistance to GHRH or other upstream regulators. His insulin-like growth factor 1 (IGF-1) level was reduced at 11.6 nmol/L (8.9 μg/dL) (13-40 nmol/L [9.9-30.6 μg/dL]) and he also had TSH resistance and extensive intracranial calcifications (5). Sano et al reported GH deficiency as documented by arginine and clonidine stimulation testing in monozygotic twins with AD-PHP1B due to a *STX16* microdeletion (4). Both patients were treated with rhGH, which improved their height SDS from −3.4 at baseline to −2.8 and −2.7, respectively, by age 8.5 years (4); final adult height data was not available. Our patient had a robust initial response after 4 months of rhGH and he continues to grow well while receiving this treatment (4).

Although rare, GHRH resistance can occur in PHP1B. Such patients should be evaluated for GHRH resistance if they present with short stature and/or reduced growth velocity, as treatment with rhGH may improve growth velocity in affected patients. Further research is needed to assess final height outcomes in youth with PHP1B treated with rhGH.

## Learning Points

- Although rare, growth hormone releasing hormone resistance (GHRH) can occur in patients with pseudohypoparathyroidism type 1B (PHP1B).
- Patients with PHP1B should be evaluated for GH insufficiency (due to GHRH resistance) if they present with short stature and reduced growth velocity.
- Treatment with recombinant human GH may improve growth velocity in PHP1B patients.

## Data Availability

Some or all datasets generated during and/or analyzed during the current study are not publicly available but are available from the corresponding author on reasonable request.

## Abbreviations

AHO: Albright hereditary osteodystrophy
AD: autosomal dominant
DMR: differentially methylated region
GH: growth hormone
GHRH: growth hormone releasing hormone
Gsα: stimulatory G protein α subunit
IGF-1: insulin-like growth factor 1
IGF-BP3: insulin-like growth factor binding protein 3
patUPD20q: paternal uniparental disomy involving chromosome 20q
PHP: pseudohypoparathyroidism
PHP1B: pseudohypoparathyroidism type 1B
PTH: parathyroid hormone
MLPA: multiplex ligation-dependent probe amplification
MS-MLPA: methylation-sensitive multiplex ligation-dependent probe amplification
rhGH: recombinant human growth hormone
TSH: thyroid-stimulating hormone
